# Light Dose is a Limiting Factor to Maintain Cell Viability in Fluorescence Microscopy and Single Molecule Detection

**DOI:** 10.3390/ijms11030956

**Published:** 2010-03-08

**Authors:** Michael Wagner, Petra Weber, Thomas Bruns, Wolfgang S. L. Strauss, Rainer Wittig, Herbert Schneckenburger

**Affiliations:** 1 Institut für Angewandte Forschung, Hochschule Aalen, Beethovenstr. 1, D-73430 Aalen, Germany; E-Mails: michael.wagner@htw-aalen.de (M.W.); petra.weber@htw-aalen.de (P.W.); thomas.bruns@htw-aalen.de (T.B.); 2 Institut für Lasertechnologien in der Medizin und Meßtechnik an der Universität Ulm, Helmholtzstr. 12, D-89081 Ulm, Germany; E-Mails: wolfgang.strauss@ilm.uni-ulm.de (W.S.L.S.); rainer.wittig@ilm.uni-ulm.de (R.W.)

**Keywords:** cell viability, light dose, fluorescence microscopy, TIR, single molecules

## Abstract

A test system for cell viability based on colony formation has been established and applied to high resolution fluorescence microscopy and single molecule detection. Living cells were irradiated either by epi-illumination or by total internal reflection (TIR) of a laser beam, and light doses where at least 90% of irradiated cells survived were determined. These light doses were in the range of a few J/cm^2^ up to about 200 J/cm^2^ depending on the wavelength of illumination as well as on the presence or absence of a fluorescent dye (e.g., the membrane marker laurdan). In general, cells were less sensitive to TIR than to epi-illumination. However, comparably high light doses needed for repetitive excitation of single molecules limit the application of super-resolution microscopy to living cells.

## 1. Introduction

In fluorescence microscopy considerable improvements in resolution have been reported in recent years. This holds in particular for confocal [1] and multiphoton [2,3] laser scanning microscopy (LSM) including 4-Pi and stimulated emission depletion (STED) microscopy, where a lateral resolution of about 30 nm as well as a high axial resolution have been obtained [4]. Those techniques, however, require exposure to very high light doses, such that damage to living cells may occur. Therefore, wide field microscopy appears to be a promising alternative, in particular since highly sensitive camera systems [5] as well as axially resolving techniques – based on structured illumination [6] or selective plane illumination microscopy (SPIM) [7] – have been introduced. Methods of super-resolution microscopy, e.g., stochastic optical resolution microscopy (STORM) or photoactivated localization microscopy (PALM), have meanwhile been reported [8–10], which commonly are based on single molecule detection, and which permit a resolution below 20 nm. However, the high light doses needed for those methods may again limit their application to living cells.

Therefore, in order to assess the limits of light exposure maintaining cell viability in fluorescence microscopy and single molecule detection, we established a test system based on colony formation of living cells. So, microscopic methods could be limited to light doses, where at least 90% of irradiated cells survived. Cells were exposed either to epi-illumination or to illumination upon total internal reflection (TIR) of an incident laser beam. While in the first case light intensity remained almost the same upon transmission of a cell layer, cell surfaces were illuminated selectively in the second case by an exponentially decaying evanescent electromagnetic field with a penetration depth of about 100 nm [11]. Due to this low penetration depth TIR also represents an optimum condition for single molecule detection [12]. Fluorescence microscopy of cultivated cells – partly incubated with a fluorescent membrane marker – and single molecule measurements of test samples are reported in the present paper.

## 2. Materials and Methods

### 2.1. Cells and Test Samples

U373-MG human glioblastoma cells were obtained from Cell Lines Service (CLS, Eppelheim, Germany, No. 300366). Cells were routinely grown in RPMI 1640 medium supplemented with 10% fetal calf serum and antibiotics at 37 °C and 5% CO_2_. In addition, U373-MG cells stably transfected with an activated TP53 suppressor gene were kindly provided by Prof. Jan Mollenhauer, Dept. of Molecular Oncology, University of Southern Denmark, Odense. Cell incubation with the fluorescent membrane marker 6-dodecanoyl-2-dimethylamino naphthalene (laurdan) [13] was performed in culture medium at a concentration of 8 μM for 1 h. For single molecule experiments highly diluted test samples of 10^−10^ M rhodamine 6G either dissolved in distilled water or embedded in agarose (1.5% in H_2_O; Fluka Chemie, Buchs, Switzerland) were used.

### 2.2. Colony Formation

**Figure 1.**
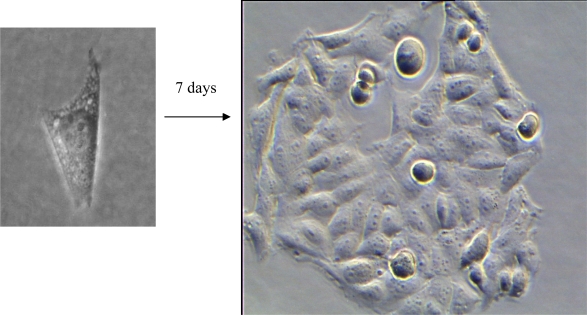
Colony formation of U373-MG glioblastoma cells after seeding of individual cells [phase contrast microscopy with 40 ×magnification; image sizes: 20 μm ×20 μm (left) and 150 μm ×105 μm (right)].

To determine cell viability, single U373-MG cells were seeded at low density (10 cells/mm^2^) in 8-well microslides (μ-Slide; Ibidi, Martinsried, Germany) and irradiated under the microscope with variable light doses and wavelengths on the 3^rd^ day after seeding. Either a continuous wave (cw) argon ion laser operated at 514 nm (Innova 90, Coherent, Palo Alto, USA) or a cw helium-neon laser operated at 633 nm (Mod. 1248, Spectra Physics, Mountain View, USA) or a laser diode emitting a quasi continuous series of picosecond pulses (LDH 375 or LDH 400 with driver PDL 800-B, Picoquant, Berlin, Germany; wavelengths: 375 nm or 391 nm; pulse energy: 12 pJ, pulse duration: 55 ps, repetition rate: 40 MHz) was used for irradiation. CW and “quasi continuous” light sources were not further distinguished with respect to cell viability. Epi-illumination of whole cells was performed in an inverted microscope (Axiovert 200, Carl Zeiss Jena, Germany), whereas for TIR illumination of thin layers a prism based setup [14] adapted to an upright microscope (Axioplan 1, Carl Zeiss Jena, Germany) was used. Spatial coordinates of irradiated cells on a positioning table (Scan IM 130×100, Märzhäuser, Wetzlar, Germany) were stored. After re-incubation of the cells with RPMI 1640 medium and further cultivation until the 7^th^ day after seeding, colony formation, as depicted in Figure 1, was evaluated. Plating efficiency, *i.e.*, the percentage of formed colonies per seeded cells, was determined, and cell survival was defined by less than 10% reduction of the plating efficiency in comparison with non-irradiated controls. For each light dose and each condition of irradiation the percentage of colony forming cells was determined after irradiation of 20 individual cells. Medians and the median absolute deviations (MADs) were calculated from four individual experiments in each case.

### 2.3. Fluorescence Microscopy

**Figure 2.**
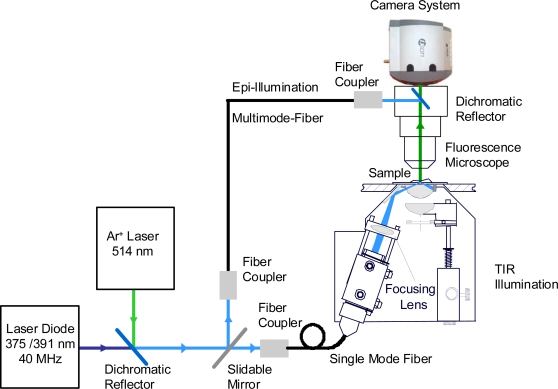
Wide field fluorescence microscope with epi- and TIR illumination as well as exchangeable camera systems (the TIR condenser includes an additional device for transillumination and phase contrast microscopy needed for adjustment of the cells).

Wide field fluorescence microscopy of cultivated cells was performed in an upright microscope (s. above) upon epi-illumination or TIR illumination by one of the laser diodes described above at a wavelength of 375 nm or 391 nm and an irradiance of 100 mW/cm^2^ (corresponding to solar irradiance). In contrast, single molecule experiments of test samples were carried out upon TIR illumination by the argon ion laser operated at 514 nm at a thousand-fold higher irradiance of 100 W/cm^2^. In this latter case the TIR condenser described in [14] was slightly modified using a focusing lens and a glass prism of extremely low intrinsic fluorescence (N-PK 51). Fluorescence images were recorded with a 40×/1.30 oil or a 63×/0.90 water immersion objective lens, an appropriate long pass filter and an electron multiplying (EM-)CCD camera with Peltier cooling; its sensitivity is below 10^−16^ W/pixel, corresponding to less than 25 photons/pixel within a time frame of 100 ms (DV887DC, ANDOR Technology, Belfast, U.K.) [5]. For some measurements of brightly fluorescent laurdan this camera was replaced by a Sony 3CCD Colour Camera (Model MC-3254, AVT Horn, Aalen, Germany) and used in combination with the software AxioVision (Carl Zeiss Jena, Germany). In addition, fluorescence spectra of single cells were recorded with a custom made polychromator and an image intensifying detection unit (IMD 4562, Hamamatsu Photonics, Ichino-Cho, Japan), whereas fluorescence decay measurements were performed with an image intensifying camera system (Picostar HR 12 image intensifier coupled to a cooled CCD camera; LaVision, Göttingen, Germany). The whole setup is depicted in Figure 2.

## 3. Results

### 3.1. Colony Formation

**Figure 3.**
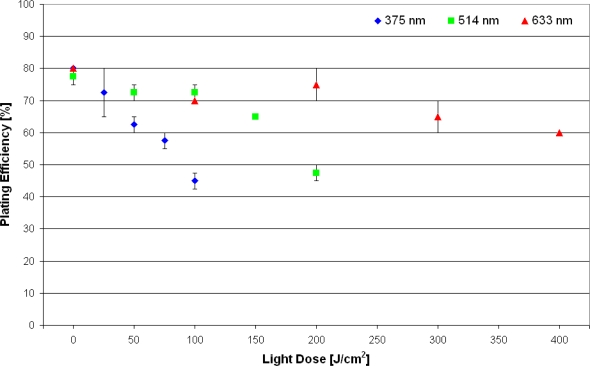
Percentage of colony formation (“plating efficiency”) of single non-incubated U373-MG glioblastoma cells upon exposure to different excitation wavelengths and light doses. Values represent medians ±MADs. The plating efficiency at 0 J/cm^2^ is defined as 100% cell survival.

**Figure 4.**
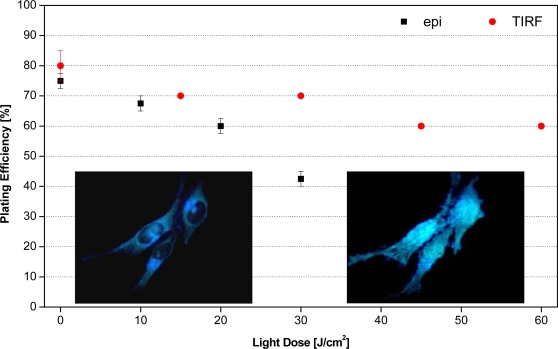
Percentage of colony formation (“plating efficiency”) of single U373-MG glioblastoma cells incubated with the membrane marker laurdan (8 μM, 1 h) upon epi-illumination of whole cells and TIR illumination of plasma membranes at 391 nm. Values represent medians±MADs. The inlays show representative fluorescence images upon illumination of whole cells (left) and plasma membranes (right); image size: 200 μm ×150 μm.

Plating efficiency of non-incubated U373-MG glioblastoma cells as a function of light dose for various wavelengths of illumination is depicted in Figure 3. While the wavelength of 375 nm appears appropriate for excitation of intrinsic fluorescence, the longer wavelengths (514 nm, 633 nm) may be used e.g., for Raman microscopy. Plating efficiency is around 80% for non-irradiated cells, but decreases upon illumination. If cell viability is defined as less than 10% reduction of the plating efficiency, cells remain viable up to a light dose of about 25 J/cm^2^ at an irradiation wavelength of 375 nm, 100 J/cm^2^ at 514 nm and about 200 J/cm^2^ at 633 nm. These doses correspond to 250 s, 1,000 s or 2,000 s of solar irradiance, respectively. Cells incubated with fluorescent dyes are generally more sensitive to irradiation, as demonstrated for the membrane marker laurdan. Upon incubation of U373-MG cells with laurdan (8 μM, 1 h) cells remained viable up to a light dose around 10 J/cm^2^ (at an irradiation wavelength of 391 nm). A higher limiting dose of about 30 J/cm^2^ was determined when only the cell surface (plasma membrane and adjacent cellular sites) was illuminated upon total internal reflection of a laser beam, as depicted in Figure 4.

### 3.2. Fluorescence Microscopy of Living Cells

**Figure 5.**
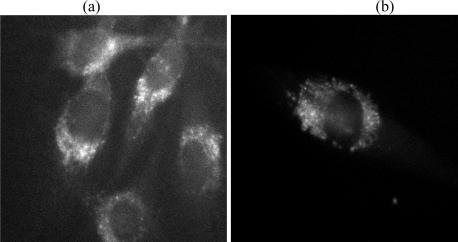
Autofluorescence of U373-MG glioblastoma cells; (a) reference cells, and (b) cells with activated TP53 suppressor gene. Excitation wavelength: 375 nm, emission measured at λ ≥ 415 nm, image size: 140 μm ×120 μm, light dose of irradiation: 0.2 J/cm^2^ each.

Autofluorescence images of U373-MG human glioblastoma cells (excited at 375 nm and measured above 415 nm) are depicted in Figure 5, and the intracellular fluorescence patterns of unaltered U373-MG cells and U373-MG cells with an activated TP53 tumour suppressor gene are compared. While the unaltered tumour cells show a partly granular and partly diffuse fluorescence pattern, the granular pattern is predominant in cells expressing the activated suppressor gene. Therefore, the evaluation of autofluorescence patterns (in addition to fluorescence spectra and lifetimes) may be a helpful tool for a classification of cancer cells and for the evaluation of tumour cell viability, e.g., in response to anticancer treatment. Light doses of about 0.2 J/cm^2^ were used for image acquisition by the highly sensitive EM-CCD camera. Even with additional detection of fluorescence spectra and decay kinetics the light dose could be limited to 10–12 J/cm^2^, and cell viability of U373-MG cells was maintained. Cell viability of U373-MG glioblastoma cells was also maintained upon incubation with the membrane marker laurdan (8 μM, 1 h), however, for recording fluorescence images, spectra and decay curves the whole “budget” of light (10 J/cm^2^) was needed upon epi-illumination. In TIR microscopy the illumination dose always remained far below the limiting dose of 30 J/cm^2^, even if about 10 variable-angle images were recorded for nanotomography of cell-substrate contacts [15].

### 3.3. Single Molecule Detection

**Figure 6.**
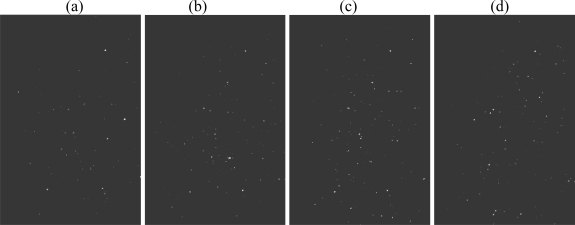

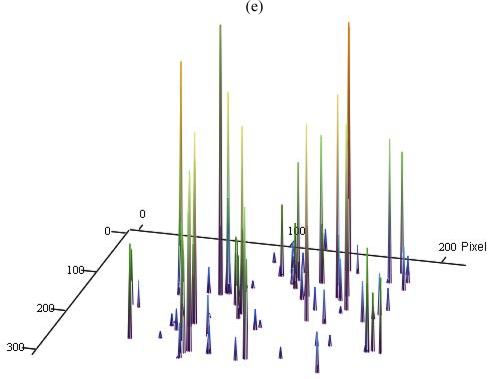
Single molecule detection of 10^−10^ M rhodamine 6G (in agarose) in 4 time intervals of 100 ms each (a–d) including a 3D plot (e) (TIR laser excitation at λ= 514 nm; fluorescence measured at λ ≥ 530 nm; image size: 60 μm ×90 μm).

Single molecule detection is based on repetitive excitation of fluorophores located at low concentration in a layer of about 100 nm thickness. To access those thin layers by light, TIR microscopy proved to be an ideal tool; however, an irradiance of about 100 W/cm^2^ (e.g., 6.5 mW of laser power applied to an area of 6,500 μm^2^) revealed to be necessary to detect a sufficient number of fluorescence photons originating from each molecule. A series of successive single molecule images (each recorded within an exposure time of 100 ms) of rhodamine 6G at a concentration of 10^−10^ M in agarose is depicted in Figure 6 together with a 3-dimensional plot. Fluorescence intensities of most molecules differed between individual images due to blinking. The illumination time of individual samples was limited to 3–10 s corresponding to a total light dose of 300–1,000 J/cm^2^.

## 4. Discussion

The light dose and wavelength of illumination proved to be essential parameters for maintaining cell viability in optical microscopy. Non-phototoxic light doses, *i.e.*, those doses where plating efficiency decreased by less than 10% in comparison with non-irradiated controls, were up to 200 J/cm^2^ (increasing with excitation wavelength) for non-incubated cells, but decreased upon application of a fluorescent dye. Photosensitization and light-induced generation of cytotoxic reactive oxygen species, e.g., singlet oxygen or superoxide radicals, has been related to various intrinsic molecules, e.g., flavins [16] or porphyrins [17,18], as well as fluorescent dyes, e.g., organelle markers or photosensitizers used in photodynamic therapy (PDT) [19]. This may explain the observed dependence of non-phototoxic light doses on excitation wavelength as well as on application of a certain fluorophore. The lowest range of non-phototoxic light doses of 0–0.25 J/cm^2^ was recently determined for the photosensitizer protoporphyrin IX (accumulation induced after incubation with 5-aminolevulinic acid (5-ALA), a precursor of porphyrin biosynthesis) [20] which corresponded to only 0–2.5 s of solar irradiation. Reduction of phototoxicity by controlled light exposure in fluorescence microscopy has been well documented in the literature [21]. Currently there is no experimental evidence that thermal damage might also affect cell viability at moderate powers of illumination used in the present study, since control measurements with an infrared camera (VarioScan, high resolution model 3021-ST; Jenoptik, Jena, Germany) did not show any temperature change above 0.2 K due to laser irradiation (633 nm; 200 J/cm^2^, data not shown).

While cell viability was generally maintained in conventional wide field microscopy with highly sensitive camera systems, additional experiments proved that the limit for non-phototoxic light doses was easily attained in axially resolved microscopy with repetitive illumination of the same samples, e.g., in laser scanning microscopy or wide field microscopy with structured illumination. Therefore, the number of selected planes has either to be limited, or individual planes have to be illuminated without light exposure of the remaining parts of the sample. This has recently been achieved by Selective Plane Illumination Microscopy (SPIM) or Light Sheet Based Fluorescence Microscopy (LSFM) [7].

Whether non-phototoxic light doses are the same upon cw and repetitive pulse excitation (e.g., in laser scanning microscopy) is still an open question. However, in all present experiments integral light dose (measured in J/cm^2^) turned out to be the main parameter for colony formation, rates of photobleaching or morphological changes. Also Murray *et al*. [22] found equal amounts of photobleaching in a wide field and a laser scanning microscope, when the total dose of illumination was the same. Studies of further phenomena, e.g., cell damage due to non-linear interactions of very short, intensive light pulses in the picosecond to femtosecond range, or cell recovery upon prolonged light exposure, were not addressed by the present experiments.

Light doses upon TIR and epi-illumination were calculated as a product of intensity and exposure time. Due to comparably low absorption and scattering within a cell layer of about 10 μm thickness, light intensity upon epi-illumination remained almost unchanged all over the cells. Upon TIR illumination the intensity I_0_ of the electromagnetic field was enhanced by a transmission factor T≈3 on the glass surface, but decreased by about the same factor at an average distance Δ≈ 100 nm between the glass substrate and the cell membrane according to the relation I = T I_0_ e^−Δ/d^, with d corresponding to the penetration depth of the evanescent wave which was again about 100 nm [14,15]. Therefore, light intensity on the cell surface corresponded to the incident intensity, but decreased rapidly within the cell. In comparison with conventional epi-illumination, cells were expected to be less sensitive to TIR illumination, where only the plasma membrane and adjacent cellular sites are exposed to light. Experimentally, non-phototoxic light doses upon TIR and epi-illumination differed by a factor of about 3 for cells incubated with laurdan (s. above) as well as for untreated cells (preliminary result, not shown). Although this factor appears rather small, lower sensitivity towards TIR illumination is e.g., advantageous in variable-angle TIR microscopy, where cell-substrate topology can be calculated with nanometer precision from a certain number (typically 10–15) of TIR images [15]. TIR microscopy is also a preferential method for single molecule detection in layers of about 100 nm thickness. In this case, however, the irradiance should be around 100 W/cm^2^ in order to absorb 10^−8^–10^−7^ W/cm^2^ and to excite each molecule several thousand times per second, such that about 100–1,000 fluorescence photons per second can be recorded. Cells incubated with very low concentrations of fluorophores may have similar sensitivity to light as non-incubated cells, *i.e.*, the maximum non-phototoxic light dose is expected to be about 100–200 J/cm^2^ upon epi-illumination and 300–600 J/cm^2^ upon TIR illumination. Therefore, the duration of a single molecule experiment should not exceed a few seconds. This should be considered in super-resolution microscopy based on single molecule techniques, e.g., STORM, PALM or single molecule based energy transfer measurements.
